# Detection and Whole-Genome Analysis of a Zoonotic G8P[14] Rotavirus Strain Isolated from a Child with Diarrhea

**DOI:** 10.1128/genomeA.01053-17

**Published:** 2017-10-12

**Authors:** Andrej Steyer, Tina Naglič, Urška Jamnikar-Ciglenečki, Urška Kuhar

**Affiliations:** a Institute of Microbiology and Immunology, Faculty of Medicine, University of Ljubljana, Ljubljana, Slovenia; b Institute of Food Safety, Feed and Environment, Veterinary Faculty, University of Ljubljana, Ljubljana, Slovenia; c Institute of Microbiology and Parasitology, Veterinary Faculty, University of Ljubljana, Ljubljana, Slovenia

## Abstract

The group A rotavirus strain SI-2987/09 was detected in a child with severe diarrhea. The whole-genome analysis revealed the G8-P[14]-I2-R2-C2-M2-A3-N2-T6-E2-H3 genome constellation, which reflects the zoonotic transmission of the strain most probably from the ungulate group. This was also confirmed by the high nucleotide identity to those animal rotavirus strains.

## GENOME ANNOUNCEMENT

Rotaviruses are still an important cause of acute gastroenteritis in children under 5 years of age. Despite the broad vaccine use since 2006, an estimated 215,000 child deaths per year are caused by the virus (1). In the postvaccine licensure period, the effectiveness of vaccination is monitored and molecular epidemiology of group A rotaviruses (RVAs) is followed to recognize possible escape mutants and the introduction of zoonotic strains into the human population. RVAs are found in various hosts, including humans and many animal species. The most common zoonotic strains detected in humans were linked to the strains detected in pigs, cows, sheep, and other ungulates (2).

Previously, we reported on zoonotic or human-animal reassortant RVA strains detected in humans, such as G4P[6], G10P[14], G8P[8], and G6P[11]. We also detected rotaviruses in cattle, pigs, and, more recently, roe deer (3–7). In this report, we present the whole-genome sequence of the RVA strain SI-2987/09, detected in a 12-month-old child, hospitalized for acute gastroenteritis with RVA as the sole detected enteric pathogen. A 10% stool suspension was processed for molecular typing, using the protocol of multiplex-nested PCR as described previously (5). Due to the failure in genotyping, the viral RNA was subjected to whole-genome analysis, using a next-generation sequencing approach on the IonTorrent PGM platform, following a previously described protocol (8).

In total, 588,814 reads were generated, and after quality checking and trimming, sequence reads were *de novo* assembled with the GS *de novo* assembler (Roche, Basel, Switzerland), generating 11 RVA genome segments with 69,774 (11.85%) used reads, giving a 937.7-fold average coverage. Open reading frames (ORFs) of the genome segments were determined with the Geneious ORF finder (Biomatters LtD., Auckland, New Zealand). An RVA genome of 18,367 nucleotides was generated. According to the Rota C genotyping tool and a BLAST search for the most identical sequences in GenBank, the RVA genotype constellation was determined as G8-P[14]-I2-R2-C2-M2-A3-N2-T6-E2-H3. Genome segment lengths in nucleotides (nt) and the most identical sequences found in GenBank were 3,302 nt for VP1 (98.2%, RVA/Human-tc/ITA/PA169/1988/G6P[14]); 2,677 nt for VP2 (99.8%, RVA/roe deer-wt/SLO/D110-15/2015/G8P[14]); 2,583 nt for VP3 (93.2%, RVA/Cow-tc/THA/A44/1989/G10P[11]), 2,352 nt for VP4 (97.2%, RVA/Human-tc/GBR/A64/1987/G10P11[14]); 1,351 nt for VP6 (96.6%, RVA/Antelope-wt/ZAF/RC-18-08/G6P[14]); 1,062 nt for VP7 (94.9%, RVA/Human-wt/HUN/182-02/2002/G8P[14]); 1,555 nt for NSP1 (99.5%, RVA/roe deer-wt/SLO/D110-15/2015/G8P[14]); 1,044 nt for NSP2 (99.5%, RVA/roe deer-wt/SLO/D110-15/2015/G8P[14]); 1,075 nt for NSP3 (95.9%, RVA/Human-wt/HUN/BP1879/2003/G6P[14]); 732 nt for NSP4 (97.8%, RVA/Human-wt/Bel/BEF06018/2014/G29P41); and 634 nt for NSP5 (98.6%, RVA/Cow-wt/SVN/SI-B17/2004/G6P[11]). The genome analysis revealed the DS-1-like genome constellation with most of the segments related to RVA found in ungulates or zoonotically transmitted strains in humans. Three of the segments were most identical to the roe deer strain of the same genome constellation found in Slovenia (7).

The detected strain SI-2987/09 shows a typical zoonotic origin and confirms a high potential for RVA zoonotic transmission between ungulates and humans. Further studies on host susceptibility to various animal rotavirus strains is needed.

### Accession number(s).

The sequences reported here were deposited in GenBank under accession numbers KY972333 (VP7), KY972331 (VP4), KY972332 (VP6), KY972328 (VP1), KY972329 (VP2), KY972330 (VP3), KY972323 (NSP1), KY972324 (NSP2), KY972325 (NSP3), KY972326 (NSP4), and KY972327 (NSP5).
